# Necrotizing Fasciitis Co-existing With Oral Squamous Cell Carcinoma: A Case Report of a Diagnostic Dilemma

**DOI:** 10.7759/cureus.62947

**Published:** 2024-06-23

**Authors:** Nikhar Wadhwani, Nitin Bhola, Pallavi Yelne

**Affiliations:** 1 Oral and Maxillofacial Surgery, Sharad Pawar Dental College and Hospital, Datta Meghe Institute of Higher Education and Research, Wardha, IND; 2 Medicine, Jawaharlal Nehru Medical College, Datta Meghe Institute of Higher Education and Research, Wardha, IND

**Keywords:** poorly differentiated squmaous cell carcinoma, infectious diseases, medical oncology, cervical necrotizing fasciitis, oral squamous cell carcinoma, necrotizing fasciitis

## Abstract

Necrotizing fasciitis is an uncommon yet highly dangerous bacterial infection characterized by rapid spread along the fascial planes and subcutaneous tissue, leading to extensive tissue necrosis and often resulting in death. The swift progression of necrosis can induce systemic sepsis, toxic shock syndrome, and multi-organ failure. While necrotizing fasciitis of the neck is rare, it typically originates from dental or pharyngeal sources. Successful treatment hinges on early diagnosis, appropriate antibiotic therapy, and surgical intervention for tissue debridement.

This article presents the case of a 40-year-old individual with necrotizing fasciitis of the neck. We herein review the clinical features, pathogenesis, and treatment approach for the case. Rapid recovery necessitated comprehensive medical treatment targeting the underlying cause with aggressive supportive measures. Surgical intervention involved thorough debridement to remove necrotic tissue, irrigation with antiseptic solutions, and early application of topical antimicrobials.

## Introduction

Necrotizing fasciitis is a rapidly progressing infection that disrupts the fascial and perifascial planes, often accompanied by penetration into skeletal muscles and deeper tissues. The characteristic soft tissue necrosis in this condition is caused by the release of proteases, exotoxins, and endotoxins which compromise microcirculation, resulting in vascular thrombosis and further exacerbating complex injuries [1-2].

Certain conditions may predispose patients to necrotizing fasciitis, including immunosuppression, diabetes mellitus, drug abuse, chronic renal disease, and malignancy. Some of the bacterial species associated with necrotizing fasciitis are also closely associated with malignancy, such as *Clostridium septicum*, which is strongly associated with colorectal cancers. It is theorized that the anaerobic glycolysis of the tumor creates a conducive environment for clostridia spores, and the disruption of the gastrointestinal mucosa by the tumor allows these spores to enter the bloodstream [3].

In recent years, a similar link has been established between various types of cancer and the oral microbiome, with a particular focus on how changes in the composition of oral bacteria contribute to oral squamous cell carcinoma (OSCC)[4]. The oral bacteria form the second-largest microbial community associated with humans, surpassed only by the bacteria in the gut. They create a unique micro-ecology in the oral cavity, where over 700 bacterial species coexist with fungi and viruses. This microflora lives harmoniously in the oral cavity unless there is a breach in the local homeostatic environment, such as the presence of a carcinoma.

We discuss a case that presented as necrotizing fasciitis, but further investigations determined the cause to be a malignancy. We address the severity and rapid dissemination of this condition, underscoring the critical importance of early diagnosis to enable prompt initiation of management.

## Case presentation

A 40-year-old male reported to the emergency department with a fever and a six-day history of swelling in the left side of his jaw extending into the neck. The patient presented with dysphagia, odynophagia, hoarse voice, and stiffness in the neck and left shoulder. Fifteen days previously the patient had noted oral ulceration with mild purulent discharge. There was no history of trauma, dental pain, or insect bites. The patient gave a habitual history of consuming chewing tobacco (in the form of *kharra* found in the Indian subcontinent) and two ounces of alcohol twice weekly for approximately the last 10 years.

**Figure 1:**
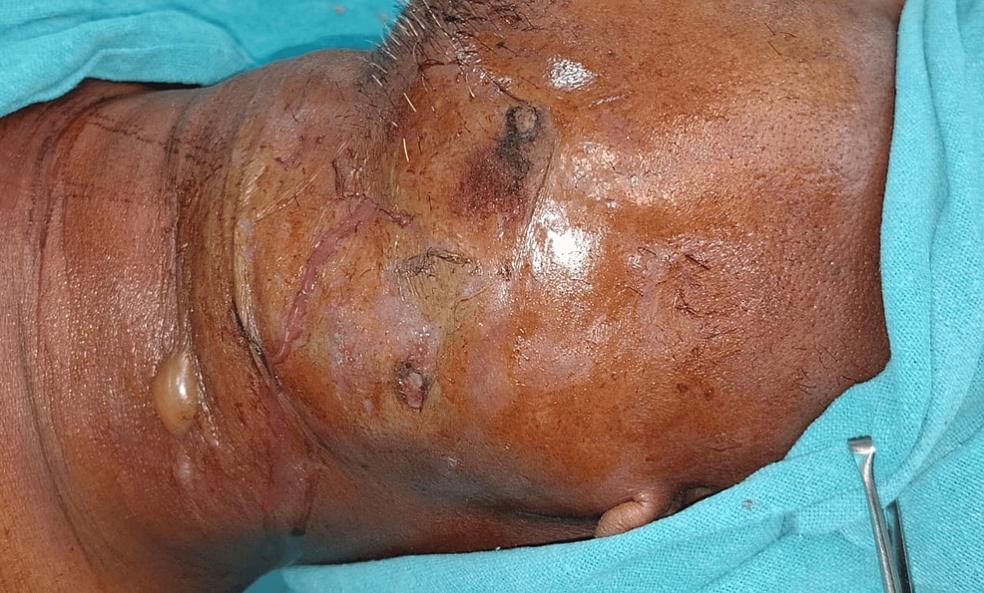
Lateral aspect of face and neck showing pink/purple discoloration of the skin, fluid discharge, and bullae

Examination revealed oxygen saturation of 91% on room air. The patient was hypotensive with a recorded blood pressure of 90/60 mmHg, heart rate of 128 beats per minute, respiratory rate of 24 cycles per minute, and a temperature of 102.4 degrees Fahrenheit. A brawny swelling of about size 6 x 4 cm was present over the left side of the jaw beginning from the corner of the mouth to the angle of the mandible posteriorly, shown in Figure 1. The swelling extended inferiorly to the left supraclavicular region, where it was associated with erythema and tenderness. Restriction of neck movements was also present. Multiple supraclavicular lymph nodes were palpable over the left side, the largest of which was about 2.5 x 2 cm in size, mobile, and tender in nature. Palpation elicited gaseous crepitus. Intraoral examination was not possible due to the presence of severe trismus.

**Figure 2:**
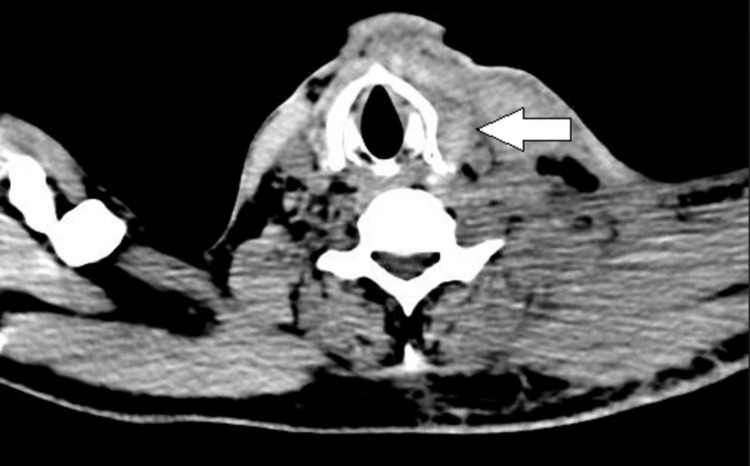
An axial cut of the computed tomography of the neck, showing compression of the laryngopharynx

**Figure 3:**
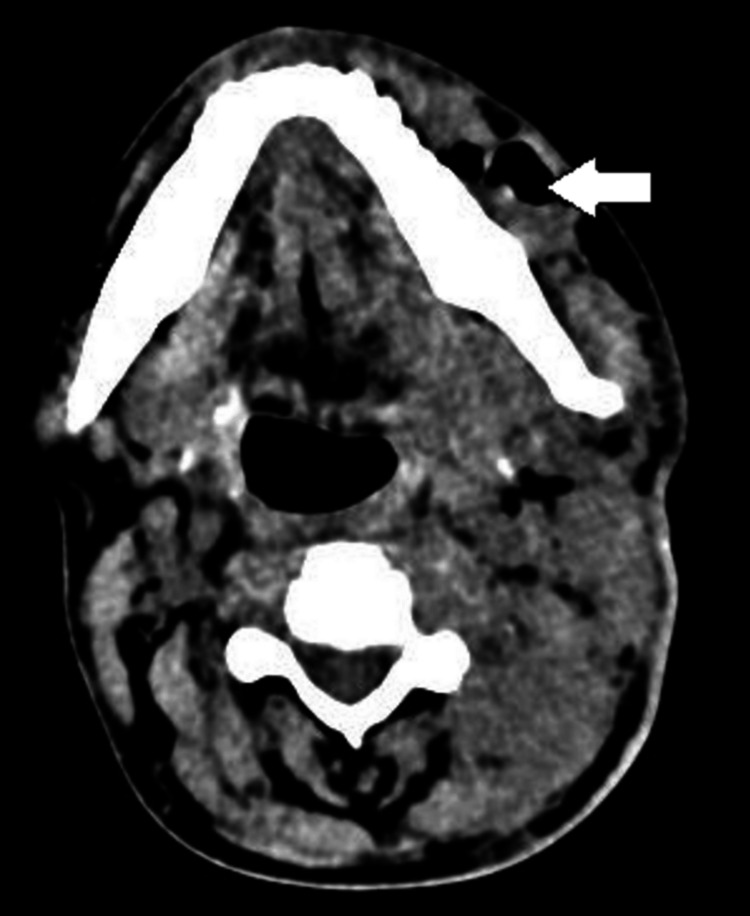
An axial section of computed tomography of the mandible, representing a radiolucent lesion over the left side

Hematological studies revealed a raised C-reactive protein (99.6), raised procalcitonin (43.1), total leucocyte count of 21000/mm^3^, and low haemoglobin 7.2 gm%. Arterial blood gases were consistent with moderate metabolic acidosis, with a pH of 7.29 and low bicarbonate levels of 17 mEq/L. The patient was not a known diabetic, but his HbA1C was 9.2, falling in the diabetic range. A contrast enhanced computed tomography (CT) scan of the head and neck confirmed the focal hypodense areas noted in left side of neck, suggesting subcutaneous edema. Compression of the laryngopharynx was noted (Figure 2). A well-defined heterogeneously enhancing lesion of size 12 x 17 mm was also noted in the left buccal mucosa (Figure 3). The blood tests and diagnostic workup of the patient are given in Table 1.

**Table 1:** Hematological studies of the patient

Blood tests and diagnostic workup	Result	Reference range
Hemoglobin	7.2 g%	12-15 g%
Mean corpuscular hemoglobin concentration	26.4%	31.5-34.5%
Mean corpuscular volume	45.4 fL	80-100 fL
Mean corpuscular hemoglobin	12 pg	27-32 pg
Total red blood cell count	6.01 million/cu mm	3.8-4.8 millions\cu mm
Total white blood cell count	21000/cu mm	4000-10000 cu mm
Platelet count	5.73 lakhs/cu mm	1.50-4.10 lakhs/cu mm
Random blood sugar	106 mg%	30-150 mg%
Urea	64 mg/dl	15-36 mg/dl
Creatinine	1.3 mg/dl	0.52-1.04 mg/dl
Serum sodium	132 mmol/L	137-147 mmol/L
Serum potassium	4.2 mmol/L	3.3-5.1 mmol/L
Serum albumin	3.7 g/dl	3.5-5.0 g/dl
Alkaline phosphatase	125 U/L	38-126 U/L
Bilirubin conjugated	0.2 mg/dl	0.0-0.3 mg/dl
Bilirubin unconjugated	0.5 mg/dl	0.0-1.1 mg/dl
Globulin	3.5 g/dl	2.4-3.5 g/dl
Serum glutamic pyruvic transaminase (SGPT)	20 U/L	<35 U/L
Serum glutamic-oxaloacetic transaminase (SGOT)	61 U/L	14-36 U/L
Total protein	7.2 g/dl	6.3-8.2 g/dl
Total bilirubin	0.7 mg/dl	0.2-1.3 mg/dl
CRP	99.6	0-5 mg/L
HbA1C	9.2	
Procalcitonin	43.1	0.07 ng/mL

Treatment and progression

The patient was admitted to the intensive care unit (ICU) with a provisional diagnosis of septic shock secondary to underlying necrotizing fasciitis. The patient received volume resuscitation measures, followed by the administration of intravenous antibiotics (piperacillin/tazobactam and metronidazole) and supportive medications. Blood cultures and local site swabs were sent, which showed the growth of Pseudomonas species and *Klebsiella pneumoniae,* respectively (Table 2). Antibiotic sensitivity reported that it was susceptible to Colistin (Table 3).

**Table 2:** Microbiology reports from blood and pus culture

Specimen	Culture
Blood	Growth of Pseudomonas species
Pus from the surgical site	Growth of *Klebsiella pneumoniae*

**Table 3:** Antibiotic sensitivity report

Organisms	Amikacin	Aztreonam	Cefipime	Ciprofloxacin	Gentamycin	Imipenem	Meropenem	Piperacillin_tazobactum	Tobramycin	Ceftazidime	Tigecycline	Amoxyclave	Ampicillin	Cefotaxime	Ceftazidime-clavulanic acid	Ceftriaxone	Colistin	Cotrimoxazole	Cefuroxime
Pseudomonas species	Sensitive	Resistant	Resistant	Sensitive	Sensitive	Sensitive	Sensitive	Sensitive	Sensitive										
Klebsiella pneumoniae	Resistant	Resistant	Resistant	Resistant	Resistant	Resistant	Resistant	Resistant		Resistant	Intermediate	Resistant	Resistant	Resistant	Resistant	Resistant	Sensitive	Resistant	Resistant

An urgent procedure was arranged in which a tracheostomy and a nasogastric tube were inserted to maintain the airway and facilitate gastric emptying. Once the patient was hemodynamically stable, two incisions were made over the left side of the neck, about 2 cm below the lower border of the mandible, that drained 20 ml of dishwater pus, pathognomonic of necrotizing fasciitis. A curved artery was introduced into the incision and pus locules were broken. Further exploration of this region revealed greying of the fascia covering the musculature, hence it was debrided and washed with hydrogen peroxide and povidone-iodine solution. Silicone drains were secured and the drained pus was sent for culture, which also reported back to have growth of *K. pneumoniae*. A biopsy of the lesion in the left buccal mucosa was performed and an intraoperative frozen section of the sample was taken, which was positive for malignancy. On further histopathological examination, a diagnosis of poorly differentiated squamous cell carcinoma was made.

Dressing of the wound was done twice daily, which consisted of flushing the drains with metronidazole and povidone-iodine solutions. The patient was deemed inoperable for malignancy due to the extent and nature of the lesion, and thus management through oral metronomic chemotherapy and palliative measures was decided. Despite the above measures, the prognosis for the patient was unfavorable. On the fifth postoperative day, he succumbed to septicemia and multiple organ failure syndrome, complications most likely secondary to advanced locoregional disease.

## Discussion

Necrotizing fasciitis, a potentially life-threatening disease, is rarely seen in the head and neck region. Early detection and prompt management are crucial since this condition can prove fatal despite aggressive treatment. Cellulitis is described as an acute pyogenic inflammation affecting the dermis and subcutaneous tissue. In contrast, necrotizing fasciitis is a more severe condition that involves the fascia and deeper subcutaneous tissues. It is characterized by intense pain, rapid spread of necrosis, and the development of gangrene in both the skin and underlying structures [5]. The organisms commonly associated with this condition include *Salmonella, Staphylococcus aureus, Bacteroides spp., Peptostreptococcus spp., Escherichia coli, *and *Klebsiella spp*. A number of systemic conditions predispose patients to necrotizing fasciitis, such as diabetes, peripheral vascular disease, renal failure, human immunodeficiency virus (HIV) infection, alcoholism, intravenous drug abuse, and those undergoing transplant surgery. In the presented case, the patient had raised HbA1C, which may indicate previously undiagnosed diabetes mellitus.

Bacterial infections are usually the primary cause of necrotizing fasciitis. In the head and neck area, it can stem from odontogenic sources in up to 50% of cases [6]. Small wounds on the skin or mucous membranes, like cuts or burns, have been recognized as potential triggers for this condition [7,8]. Additionally, cases stemming from a parotid abscess and post-multimodal cancer treatment have been reported [9]. There are very few cases in the literature that report necrotizing fasciitis arising from OSCC. A few incidences of necrotizing fasciitis developing from vulvar carcinoma have been reported [10], and the incidence of such a cascade is rare in the head and neck region, but not completely abstruse.

Necrotizing fasciitis is categorized into two types. Type I is a synergistic polymicrobial infection involving both aerobes and anaerobes, commonly found in patients with diabetes, peripheral vascular disease, and postoperative conditions. The bacteria responsible for Type I include *S. aureus, Streptococci, Enterococci, E. coli, Bacteroides fragilis*, and *Clostridia*. Type II is a monomicrobial infection caused by group *A *Streptococci* (Streptococcus pyogenes)* and *Methicillin-Resistant S. aureus* (MRSA) and is observed in patients without underlying comorbidities.

A number of bacteria presumed to precipitate the development of OSCC overlap with the spectrum of microbes associated with necrotizing fasciitis. During the development of OSCC, different bacteria exhibit distinct alterations. Some bacteria show significantly higher abundance in OSCC patients, while others are less prevalent in OSCC tissues and more common in healthy samples. Based on these compositional changes, certain combinations of bacteria are considered potential markers for oral cancer diagnosis. The development of OSCC involves mechanisms such as accelerated cell proliferation, evasion of immune surveillance, invasion and metastasis, inhibition of intrinsic apoptosis, promotion of angiogenesis, and support for cancer stem cells. Specific oral bacteria like *Porphyromonas gingivalis* and *Fusobacterium nucleatum* participate in many of these pathways, aiding OSCC development [3]. Additionally, inflammation plays a role in this process, with inflammatory cytokines such as interleukin (IL)-6, IL-8, tumor necrosis factor (TNF) -α, and Prostaglandin (PGE) affecting the tumor microenvironment and potentially promoting tumor progression [11].

The mainstay treatment for necrotizing fasciitis continues to be prompt and aggressive debridement along with broad-spectrum antibiotic therapy. Systemic antibiotic treatment typically includes an aminoglycoside, a penicillin, and clindamycin. Furthermore, hyperbaric oxygen therapy, in conjunction with surgery and antibiotic treatment, has been demonstrated to decrease morbidity and mortality rates. 

Poorly differentiated squamous cell carcinoma is known to have a worse prognosis than its well-differentiated and moderately differentiated counterparts, with the five-year overall survival rates being 30% lower [12]. Patients with the poorly differentiated subtype are more prone to local recurrence or distant metastasis (DS), exhibiting markedly distinct characteristics compared to well-differentiated SCC [13]. Moreover, the resection of T3-T4 tumors of this histology requires resection margins of 2 cm or more to ensure oncologic safety, which was not possible in our case due to an already large lesion, and hence palliative care with oral metronomic chemotherapy was recommended.

## Conclusions

OSCCs are still highly prevalent in the South Asian region, and the decreased immunity associated with malignancy gives rise to a number of other opportunistic infections. Wounds arising from oral carcinomas may pave the path for a more deadly infection that may spread into deeper tissue. It is important to include necrotizing fasciitis as a differential diagnosis when encountered with such an atypical and non-specific patient presentation so that rapid assessment and management can be undertaken.
